# Evaluation of the result of single-incision laparoscopic surgery for gastrointestinal stromal tumors in the stomach

**DOI:** 10.1186/s40792-019-0605-x

**Published:** 2019-03-29

**Authors:** Takashi Tatara, Shingo Kanaji, Satoshi Suzuki, Ryo Ishida, Hiroshi Hasegawa, Masashi Yamamoto, Yoshiko Matsuda, Kimihiro Yamashita, Taro Oshikiri, Takeru Matsuda, Tetsu Nakamura, Yasuo Sumi, Yoshihiro Kakeji

**Affiliations:** Division of Gastrointestinal Surgery, Department of Surgery, Graduate School of Medicine, Kobe University, 7-5-2 Kusunoki-cho, Chuo-ku, Kobe, Hyogo 650-0017 Japan

**Keywords:** Submucosal tumor, Gastrointestinal stromal tumor, Laparoscopic gastric resection, Single-incision laparoscopic surgery, Tumor location

## Abstract

**Background:**

Single-incision laparoscopic surgery (SILS) has recently been used for the management of gastrointestinal stromal tumors (GIST). Here, the feasibility and effectiveness of SILS for GIST and indications for SILS according to tumor location were investigated.

**Case presentation:**

From July 2009 to May to 2013, a total of 14 patients underwent SILS for GIST. In 14 patients, 5 patients had tumor near the esophagogastric junction, 4 patients on the lesser curvature, 2 patients on the anterior wall, 2 patients on the posterior wall, and 1 patient on the greater curvature. The surgery of one patient with lesser curvature tumor was converted to conventional laparoscopic surgery because of technical difficulties. Another patient required re-operation because of a posterior wall tumor causing gastric obstruction. There was no complication in patients with tumors on the anterior wall and greater curvature.

**Conclusions:**

Because SILS for GISTs located mainly on the anterior wall was feasible, SILS may be considered the most appropriate type of laparoscopic surgery for GISTs in this location. However, for GISTs on the posterior wall or with lesser curvature, which require more complex management, SILS is challenging and should be carefully adapted.

## Background

Since the development of endoscopic ultrasonography and fine-needle aspiration, gastric submucosal tumors (SMTs), including asymptomatic tumors of less than 1 cm, have been more frequently detected. Gastrointestinal stromal tumors (GISTs) are potentially malignant tumors, which are often treated by surgical resection. Because GISTs rarely involve the lymph node and require a resection margin that is only grossly negative, laparoscopic gastric resection has become an acceptable option [1, 2]. The National Comprehensive Cancer Network and the Japanese Clinical Practice Guidelines on GIST recommend that laparoscopic resection, conducted by an expert laparoscopic surgeon, may be suitable for tumors of 5 cm or less in diameter [3, 4]. Furthermore, the use of single-incision laparoscopic surgery (SILS) for GISTs has recently become more common [5, 6]. However, there are only few reports describing SILS for GISTs, in particular, modification of the SILS procedure depending on tumor location [7].

Previously, we demonstrated that laparoscopic surgery was effective for SMTs adjacent to the esophagogastric junction (EGJ) [8]. In this study, the feasibility and effectiveness of SILS for GISTs was evaluated, and the need to adapt SILS according to tumor location is discussed.

## Case presentation

### Patients

A retrospective review was performed using data from 14 patients who underwent SILS for GIST at Kobe University Hospital between July 2009 and May 2013. The indicative criterion for laparoscopic surgery was a lesion diameter of less than 5 cm. Patients’ background characteristics and surgical, clinicopathological, and follow-up data were collected from medical records. All patients were operated by endoscopic surgical skill qualification system of the Japan Society of Endoscopic Surgery qualified surgeons. This study was approved by the Institutional Review Board of Kobe University.

### Procedural techniques for SILS

**Fig. 1:**
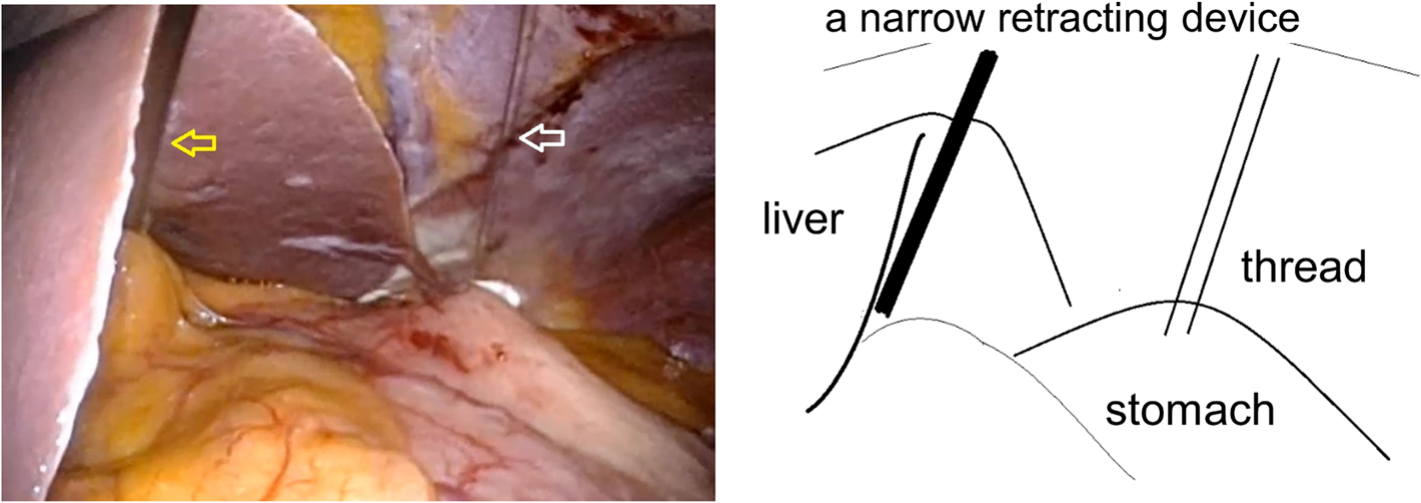
The way of our keeping the operative field. A suture was placed across the gastric wall at the anal side of the tumor, and the thread was pulled out from the abdominal cavity (white arrow). The left lateral segment of the liver was elevated with a narrow retracting device (yellow arrow).

During SILS, the surgeon stood between the legs of the patient. A round or oval Lap Protector device (Hakko Co. Ltd., Tokyo, Japan) was placed through a single 25-mm longitudinal umbilical incision, and a round or oval E.Z Access device (Hakko Co. Ltd) was placed over the Lap Protector. A 5-mm trocar was then inserted laterally to the left, and a 12-mm trocar was inserted to the right, into the E.Z Access device. A 5-mm flexible scope was inserted through the 5-mm trocar at the extreme caudal position of the E.Z Access port for the duration of the procedure. In all patients, intraoperative endoscope was used, the linear stapler was used for both resection and suture, and extraluminal approach and open type resection was performed. To keep the operative field, we constructed the following devices. A suture was placed across the gastric wall at the anal side of the tumor, and the thread was pulled out from the abdominal cavity to enhance the operative field. A second method to prevent the liver obscuring the operating field was to raise the left lateral segment of the liver with a narrow retracting device (Fig. 1).

### Patient characteristics and operative outcomes

The characteristics and operative outcomes of the study population are summarized in Table 1. A total of 14 patients underwent SILS for GIST. In 14 patients, 5 patients had tumor near the esophagogastric junction (EGJ), 4 patients on the lesser curvature, 2 patients on the anterior wall, 2 patients on the posterior wall, and 1 patient on the greater curvature. The term “located near the EGJ” was defined as “within 3 cm of the EGJ based on the preoperative endoscopic measurement”. The surgery of one patient with lesser curvature GIST was converted to conventional laparoscopic surgery due to technical difficulties. One patient who had a tumor on the posterior wall developed gastric obstruction and required distal gastrectomy. No complications were reported in patients who had a tumor on the anterior wall or greater curvature. There were no recurrences in the follow-up period.

**Table 1:** Characteristics and surgical outcomes of patients

Age (years)	Sex	Year of operation	BMI	Tumor size (mm)	Location	Developmental pattern	Operative time (min)	Complication
68	M	2009	20.6	30	Pos	Extra	192	Non
70	F	2009	17.9	33	NEJ	Intra	191	Non
67	M	2010	31	40	NEJ	Intra	189	Non
50	F	2010	20.3	37	NEJ	Intra	195	Non
57	M	2010	27.2	27	Gre	Extra	125	Non
49	F	2010	19.5	35	Les	Extra	102	Non
54	F	2010	23.8	30	NEJ	Intra	236	Non
63	M	2011	22	20	Ant	Intra	113	Non
40	F	2011	19.5	35	Les	Extra	100	Non
79	M	2011	19	28	Pos	Extra	467	Obstruction
64	F	2011	15.8	32	Les	Intra	185	Non^a^
70	F	2012	20	23	Les	Extra	76	Non
67	M	2013	24.7	16	Ant	Intra	86	Non
73	F	2013	21.1	19	NEJ	Intra	137	Non

*NEJ* near esophagogastric junction, *Ant* anterior wall, *Pos* posterior wall, *Les* lesser curvature, *Gre* greater curvature, *Extra* extraluminal, *Intra* intraluminal

^a^Conversion to conventional laparoscopic surgery

## Discussion

We reported the outcomes of 14 patients who underwent SILS for GIST.

In our reports, though all patients were operated by endoscopic surgical skill qualification system of Japan Society of Endoscopic Surgery qualified surgeons, operative time was longer than other reports from Japan and other countries [6, 7]. Recent development of surgical devices such as the three-dimensional monitor and linear stapler may enable us to shorten the operative time.

SILS has some advantages such as better cosmetic outcomes because the use of trans-abdominal ports results in fewer wounds. The cosmetic benefit of SILS for GIST resections, which leaves only a single umbilical scar, may increase patient satisfaction [9, 10]. Deveci et al. reported that patients who underwent SILS for cholecystectomy had higher cosmetic satisfaction relative to those who underwent laparoscopic surgery involving three ports [11]. Furthermore, Ceci et al. reported that SILS for appendectomy was associated with a lower risk of postoperative wound infection [12]. Considering the effectiveness of SILS, it has the potential to become the first choice for GISTs, providing that technical difficulties are surmounted.

On the other hand, SILS has some disadvantages, including conflicts between the laparoscope and instrument, limited movement of instruments, limited organ retraction, and difficulty maintaining the operative field [7]. To address these problems, we constructed the following devices. A suture was placed across the gastric wall at the anal side of the tumor, and the thread was pulled out from the abdominal cavity to enhance the operative field. A second method to prevent the liver obscuring the operating field was to raise the left lateral segment of the liver with a narrow retracting device (Fig. 1).

In conventional laparoscopic surgery, the linear stapler is inserted from the left or right side of the port, whereas in SILS, it is only inserted from the umbilical port. However, stapler insertion may be difficult if the transumbilical space is overcrowded with instruments. Attempts to adjust the resection line by moving the stapler are often unsuccessful. As an alternative, Takata et al. recommended the “move the ground” technique, in which the lesion is brought toward the stapler using an articulated grasper [7]. Despite these improvements, challenges in SILS are often related to the location of the tumor, for example, the need for complicated manipulation by the operator’s left forceps to turn over the posterior wall. In the present study, although there were only two patients with GIST on the posterior wall, gastric obstruction following re-operation occurred in one of the two cases. According to the postoperative finding by computed tomography and a contrast medium, we considered the deformation of the stomach occurred due to twisted resection by staple. Stapler use was restricted, and assistance from the left forceps was necessary to turn the gastric posterior wall, which may have caused this severe local complication. In using stapler on tumors on the posterior wall or lesser curvature, handling of the stapler is sometimes difficult. Therefore, we should take care of the resection line not to lead to deformation of the stomach, considering the technical difficulties; SILS for GIST on the posterior wall should be carefully adapted.

In a previous study, we reported the safety of SILS for gastric SMTs near the EGJ, which is challenging due to the risk of EGJ narrowing [8]. During SMT resections, endoscopic observations were used to maintain a sufficient margin from the mucosal side. Furthermore, by opening the lumen of the EGJ using the endoscope as a bougie, the gastric obstruction was prevented. Surgical outcomes of SILS for GISTs located mainly on the anterior wall did not include severe complication. However, because the gastric antrum is narrower than the cardia, it was not possible to adapt SILS in the three cases with GISTs near the pylorus ring. In specific cases, for example, when the tumor location is anterior, SILS may be a safe option for GISTs near the pylorus ring if performed by an experienced surgeon.

Intraluminal tumor is sometimes more difficult than extraluminal tumor because adding seromyotomy and bringing tumor outside of the gastric wall were necessary before resection. As we showed in Table 1, though we could resect the intraluminal tumor as safely as the extraluminal tumor, we took relatively long operative time in SILS of intraluminal tumor compared with that of extraluminal tumor, which may indicate the difficulty of SILS for intraluminal tumor. Considering these results, SILS on extraluminal tumor may be feasible; on the other hand, other operative methods such as laparoscopic and endoscopic cooperative surgery may be superior to SILS for intraluminal tumor.

## Conclusion

Based on safety and cosmetic satisfaction, SILS for GIST may become the first choice, especially for tumors located mainly on the anterior wall, including tumors near the EGJ. However, SILS for tumors located on the posterior wall should be carefully adapted due to the necessity for complicated manipulation of the stomach, which may cause injury.

## Abbreviations

EGJ: Esophagogastric junction
GIST: Gastrointestinal stromal tumor
SILS: Single-incision laparoscopic surgery
SMT: Submucosal tumor
